# Clear Cell Adenocarcinoma of Uterine Cervix: A Single Institution Retrospective Experience

**DOI:** 10.3389/fonc.2020.532748

**Published:** 2020-11-13

**Authors:** Zhimin Liu, Junyun Li, Haifeng Gu, Hua Tu, Guochen Liu, Jihong Liu

**Affiliations:** ^1^Department of Gynecologic Oncology, State Key Laboratory of Oncology in South China, Collaborative Innovation Center for Cancer Medicine, Sun Yat-sen University Cancer Center, Guangzhou, China; ^2^Department of Radiation Oncology, State Key Laboratory of Oncology in South China, Collaborative Innovation Center for Cancer Medicine, Sun Yat-sen University Cancer Center, Guangzhou, China

**Keywords:** cervix, clear cell carcinoma, surgery, radiotherapy, prognosis

## Abstract

**Purpose:**

The purpose of the study is to summarize the clinical characteristics and identify the prognosis of clear cell adenocarcinoma of the uterine cervix (CCAUC) in patients without a history of diethylstilbestrol (DES) exposure.

**Methods:**

Forty-two patients with CCAUC, treated initially at Sun Yat-sen University Cancer Center between 1985 and 2017, were studied.

**Results:**

Of all the CCAUC patients, the median age was 47 years old, and the median tumor size was 3 cm. Thirty-four early stage patients (IB = 28, IIA = 6) underwent radical surgery. Eight advanced stage patients (IIB = 8) received concurrent chemoradiotherapy (n = 4) or radical surgery (n = 4). Survival analysis showed that patients with early stage (IB-IIA) had a significantly better 5–year progression-free survival (PFS) and overall survival (OS) than those with advanced stage (IIB) (*p *< 0.05). The patients with negative pelvic lymph node (PLN) had a significantly better 5-year PFS and OS than those with positive PLN (*p *< 0.05). Radiotherapy (RT) did not affect PFS or OS in early stage patients with intermediate risk factors (*p *> 0.05). Adjuvant chemotherapy (CT) did not affect PFS or OS in early stage patients without risk factors (*p *> 0.05).

**Conclusion:**

The FIGO stage and pelvic node status were important prognostic factors for both PFS and OS. For treatment modality, we recommended that radical surgery alone was used in early stage patients without high risk factors. Ovarian preservation in early stage patients involved some risk.

## Introduction

Clear cell adenocarcinoma of the uterine cervix (CCAUC) is extremely rare among the cervical cancer, which accounts for 4% to 9% of all adenocarcinomas of the uterine cervix (1). Nevertheless, in the United States and the Netherlands, many women who were *in utero* diethylstilbestrol (DES) exposed developed a clear cell adenocarcinoma of the vagina and cervix. Among the DES-related women, the estimated incidence of CCAUC from birth to 39 years old is 1.6 per 1,000 women (2). The highly significant association between *in utero* exposure to DES and subsequent development of CCAUC in the young women was shown in a case series study in 1971 (3). With the ban of DES, DES-associated clear cell adenocarcinoma of the uterine cervix is observed much less frequently. Recently, more and more attention is paid to the non-DES-associated CCAUC (4–6).

Because of the low incidence of CCAUC, there are very limited data about the clinical behavior, pathology characteristics, optimal management, the patterns of metastasis and recurrence, and prognosis about this disease. Therefore, the large sample research of CCAUC is very meaningful.

The aim of our investigation is to summarize the clinical characteristics and identify the prognosis, through analyzing our single cancer center patients who were diagnosed with CCAUC without a history of DES exposure.

## Methods

A retrospective review was conducted at the Sun Yat-sen University Cancer Center (SYSUCC) from 1985 to 2017. All patients who were confirmed to be CCAUC were treated in our hospital. No patient had a history of DES exposure. The pathology review was conducted by two pathologists in our center. The complete medical records, including demographic, patients’ characteristics, treatment, and follow-up were collected detailedly. The 2009 and 2018 International Federation of Gynecology and Obstetrics (FIGO) staging criteria of cervical cancer were used for all patients’ staging, respectively.

The primary treatment included radical surgery or radiotherapy (RT). Surgical treatment consisted of different types of hysterectomy with or without salpingo-oophorectomy plus pelvic lymphadenectomy (PLD) with or without para-aortic lymphadenectomy; all surgical approach was laparotomy. Whether to perform para-aortic lymphadenectomy depended on imaging examination, operative exploration, and discretion of the attending surgeon. Postoperative adjuvant therapy (adjuvant radiotherapy or chemotherapy) depended on risk factors, multidisciplinary team (MDT), and our institutional practices at that time.

The follow-up schedule was included every 3 months in the first 2 years, then twice yearly for 3 years, and then once yearly. The follow-up period was defined as the time interval between the date of surgery (or diagnosis in nonsurgical patients) and either the date of death or the latest date of confirmed survival. Patients who had previous malignant disease, died of a cause not related to cervical cancer, were excluded.

The survival time, including overall survival (OS) and progression-free survival (PFS), was defined as from the date of surgery to the date of death or final clinical follow-up and the date of recurrence, respectively.

The SPSS statistical software package version 16.0 (IBM Corporation, Armonk, NY, USA) was used for all analyses. Kaplan–Meier and log-rank tests were used for survival analysis. P values less than 0.05 were considered statistically significant.

### Ethics Approval

This study was approved by the Sun Yat-sen University Cancer Center Research Ethics Committee. All methods were performed in accordance with the guidelines and regulations of this ethics board. In accordance with the ethical approval, informed consent was not required due to this being a historical material, so the Hospital Ethics Committee agreed to the informed consent waiver.

## Results

We retrospectively analyzed the data of 42 patients with CCAUC. The median age of the eligible patients was 47 years (range, 20 –78 years). Three patients were asymptomatic and diagnosed at routine physical examination (3/42, 7.0%), while other patients presented with obvious symptoms including abnormal vaginal bleeding (36/42, 86.0%) and vaginal discharge (3/42, 7.0%). All patients had abnormal-looking cervixes including exophytic lesions (25/42, 59.5%) and endophytic lesions (or barrel-shaped cervixes, 17/42, 40.5%). High risk HPV (hrHPV) test was performed in 19 patients through the HC2 method, hrHPV-positive was identified in 5 patients (26.3%), and hrHPV-negative was identified in 14 patients (73.7%). The median tumor size was 3 cm (range, 1–9 cm). The distribution of 2009 FIGO stage was as follows: stage IB-IIA, 81.0% (n = 34; IB1 = 19, IB2 = 9, IIA1 = 2, IIA2 = 4), and stage IIB, 19.0% (n = 8). The distribution of 2018 FIGO stage was as follows: stage IB-IIA, 69.0% (n = 29; IB1 = 5, IB2 = 12, IB3 = 7, IIA1 = 2, IIA2 = 3), and stage IB-IIIC, 31.0% (n = 13; IIB = 6, IIIC1 = 6, IIIC2 = 1). The different clinicopathological characteristics of all patients are summarized in **Table 1**.

**Table 1:** Clinical and pathological characteristics of the patients with CCAUC (n=42).

**Age, median (range), y**	47 (20–78)
**Symptoms, n (%)**	
Irregular vaginal bleeding	36 (86.0%)
Abnormal vaginal discharge	3 (7.0%)
No symptoms	3 (7.0%)
**HPV status**	
Positive	5 (26.3%)
Negative	14 (73.7%)
Unknown	23
**Tumor size, median (range), cm**	
≤4	26 (61.9%)
≥4	16 (38.1%)
**FIGO stage (2009)**	
IB-IIA	34 (81.0%)
IIB	8 (19.0%)
**FIGO stage (2018)**	
IB-IIA	29 (69.0%)
IIB-IIIC	13 (31.0%)
**Treatment**	
Surgery	38 (90.5%)
Radiotherapy	4 (9.5%)
**Abnormal-looking cervixes**	
Exophytic lesions	25 (59.5%)
Endophytic lesions	17 (40.5%)
**PLN metastasis**	
Yes	7 (18.9%)
No	30 (81.1%)
Unknown	7
**Para-aortic lymph nodes metastasis**	
Yes	1 (9.1%)
No	10 (90.9%)
Unknown	31
**Deep stromal invasion**	
Yes	15 (39.5%)
No	23 (60.5%)
Unknown	4
**LVSI**	
Yes	5 (13.2%)
No	33 (86.8%)
Unknown	4
**Ovarian metastasis**	
Yes	1 (3.2%)
No	30 (96.8%)
Unknown	11
**Parametrium or surgical margin involvement**	
Yes	1 (2.6%)
No	37 (97.4%)
Unknown	4

FIGO, International Federation of Gynecology and Obstetrics; LVSI, lymphovascular space involvement.

Thirty-eight patients underwent radical hysterectomy. Pelvic lymphadenectomy was performed in 37 patients, yielding a median of 24 nodes (range, 9–51 nodes). Para-aortic lymphadenectomy was performed in 11 patients (29.7%), yielding a median of six nodes (range, 1–10 nodes). Thirty-one patients (81.6%) underwent bilateral salpingo-oophorectomy. Pathological examinations after surgery were as follows: pelvic lymph nodes metastasis in 7 patients (18.9%), para-aortic lymph nodes metastasis in 1 patient (9.1%), deep stromal invasion in 15 patients (39.5%), lymphovascular space involvement (LVSI) in 5 patients (13.2%), parametrium or surgical margin involvement in 1 patient (2.6%), and ovarian metastasis in 1 patient (3.2%).

Seven patients with high risk factors (lymph node metastases, parametrium or surgical margin involvement) received concurrent chemoradiotherapy (CCRT). Only intermediate risk factors [lymphovascular space involvement (LVSI), deep stromal invasion, or tumor size beyond 4 cm] were identified in 17 patients; 12 received adjuvant radiotherapy (RT) with or without chemotherapy (CT). Survival analysis revealed that RT did not affect PFS or OS in patients with early stage with intermediate risk factors (*P > 0.05*). Among 14 patients without risk factors, 7 received chemotherapy (CT) alone, and 7 received no further treatment. The chemotherapy regimens consisted of platinum (cisplatin or carboplatin) with/without paclitaxel. Survival analysis revealed that adjuvant chemotherapy did not affect PFS or OS in patients with early stage without risk factors (*P > 0.05*).

Recurrence occurred in 11 of all CCAUC patients; the median time to recurrence was 19 months (range 8–56). Recurrences were identified in six patients with CCAUC (IB to IIA), three had pelvic recurrences, two had distant recurrences. Interestingly, one patient with stage IB1 disease who was performed with radical hysterectomy and pelvic lymphadenectomy, without risk factors after surgery, had metastasis of the left ovary at 56 months, and was performed with bilateral salpingo-oophorectomy and then given six cycles of chemotherapy consisting of cisplatin 65–70 mg/m^2^ and paclitaxel (135–170 mg/m^2^). Now, the patient is still alive with no evidence of recurrence at follow-up time for 80 months.

Eight patients were diagnosed with stage IIB disease: four received concurrent platinum-based chemoradiotherapy, and four received neoadjuvant chemotherapy (NACT) followed by radical surgery, which included two or three cycles of platinum (Cisplatin: 65–75 mg/m^2^ or carboplatin: AUC5-6) with paclitaxel (135–175 mg/m^2^) chemotherapy regimens 3 weeks before surgery. Four patients experienced pathologic down staging on surgical specimens. There were 5 recurrences among those patients: two patients received radical surgery, and three patients received radical radiotherapy.

**Figure 1:**
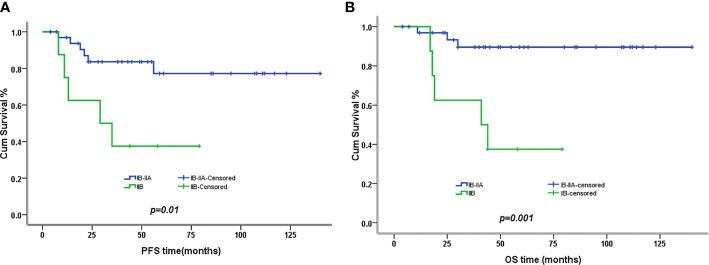
Kaplan–Meier plot of progression-free (PFS) and overall survival (OS) of different stages (2009 FIGO staging criteria). **(A)** The 5-year PFS of patients with stages IB to IIA and stage IIB was 77% versus 37% (*p *= 0.01), respectively. **(B)** The 5-year OS of patients with stages IB to IIA and stage IIB was 90% versus 37% (*p *= 0.001), respectively.

**Figure 2:**
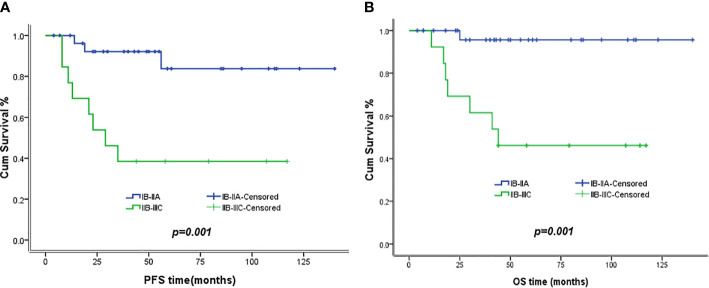
Kaplan–Meier plot of PFS and OS of different stages (2018 FIGO staging criteria). **(A)** The 5-year PFS of patients with stages IB to IIA and stages IIB to IIIC was 83.8% versus 38.5% (*p *= 0.01), respectively. **(B)** The 5-year OS of patients with stages IB to IIA and stages IIB to IIIC was 95.7% versus 46.2% (*p *= 0.001), respectively.

**Figure 3:**
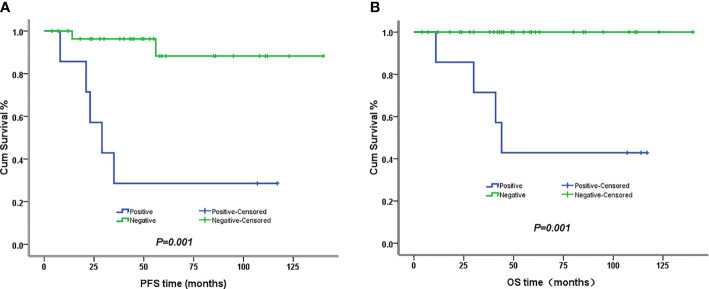
Kaplan–Meier plot of PFS and OS of different lymph node status. **(A)** The 5-year PFS of patients with and without lymph node involvement was 29% versus 88% (*p *= 0.001), respectively. **(B)** The 5-year PFS of patients with and without lymph node involvement was 43% versus 100% (*p *= 0.001), respectively.

The 5-year PFS and OS for all of the CCAUC patients were 68.5% and 77.3%, respectively. In the 2009 FIGO staging criteria, the 5-year OS for stage IB to IIA and stage IIB was 89.6% and 37.5%, respectively. Survival analysis showed that patients with stage IB-IIA CCAUC had a significantly better 5-year PFS and OS than those with stage IIB (*p < 0.05*) (**Figure 1**). In the 2018 FIGO staging criteria, the 5-year OS for stage IB to IIA and stage IIB-IIIC was 95.7% and 46.2%, respectively. Survival analysis showed that patients with stage IB-IIA CCAUC had a significantly better 5-year PFS and OS than those with stage IIB-IIIC (*p < 0.01*) (**Figure 2**). The patients with negative PLN had a significantly better 5-year PFS and OS than those with positive pelvic lymph node (PLN) (*p < 0.05*) (**Figure 3**). Tumor size(>4 cm), deep stromal invasion, and lymphovascular space involvement (LVSI) did not affect PFS or OS (*p > 0.05*).

## Discussion

CCAUC is a rare histologic subtype of adenocarcinomas of the uterine cervix, which accounts for 4% to 9% of all cervical adenocarcinomas (7). *In utero* exposure to DES was a known predisposing factor of CCAUC, since several DES-exposed cohorts’ reports from the United States and the Netherlands (2, 3, 8). Recently, much more non-DES-associated CCAUC had been reported after the ban on DES. However, the reports contained only a few case reports and case series with a small number of patients. It is unclear about the clinical characteristics and treatment recommendations of patients with CCAUC.

A bimodal age distribution of patients without exposure to DES, with one peak at 26 years and the other peak at 71 years, had been observed among the patients in the Netherlands, which suggested that CCAUC might tend to affect menarche and menopause women (2). In Jiang et al.’s study (4), which included 32 patients without DES exposure, they also identified a bimodal distribution; at the same time, the median age was 38 years. They concluded that adolescents and young women might develop into CCAUC more than other types of cervical adenocarcinoma. Thomas et al.’s (5) and Yang L et al.’s studies (6), which included 34 and 47 patients without utero DES exposure, revealed that the median age of all patients was 53 and 52 years, respectively, without separated peaks, which showed that CCAUC often affected elder women in the post-DES era. A retrospective study from Austria (1), which included 15 CCAUC without a history of *in utero* exposure to diethylstilbestrol (DES), 444 squamous cell carcinomas (SCC), and 59 non-clear cell adenocarcinomas, the age distribution of patients with CCAUC was similar to that of the other groups; the median age was 47 years. In our study, the median age at the time of diagnosis was 47 years. Our finding was consistent with Austria’ study (1) and showed that the age of onset in CCAUC patients without exposure to DES was similar to cervical SCC patients.

Adenocarcinoma of the cervix has increased over the past decades, probably because cytologic screening is less effective for adenocarcinoma. Screening methods with the hrHPV test might raise the detection of adenocarcinoma (9, 10). Thomas et al. (5) reported that abnormal Pap smear was only noted in 6 (18%) of 31 patients, which concluded that a lower frequency of abnormal cervical cytology was identified in CCAUC. Not only that, this tumor seems to be unrelated to hrHPV infection. Goto et al. (11) reported a case combination of SCC and clear cell adenocarcinoma, whose hrHPV was found in SCC, but no HPV was detected in CCAUC. Kocken M et al. (12) reported that hrHPV seemed not involved in DES-related and of limited importance in non-DES-related CCAUC, through testing for hrHPV of 28 women diagnosed with CCAUC by two PCR methods. In our study, HPV positive was noted in 5 of 19 patients (26.3%). So, as an uncommon histological type of adenocarcinoma of the cervix, CCAUC may be difficult to diagnose early because of the lack of effective screening methods.

Clinically, CCAUC could be asymptomatic or could present with abnormal vaginal bleeding or vaginal discharge, which was similar to other types of adenocarcinoma and the common squamous cell carcinoma (4–6, 13, 14). Reich O et al. (1) reported that clear cell adenocarcinoma of the uterine cervix (CCAUC) showed predominantly endophytic lesions (80%) and extended to the uterine corpus significantly more often than squamous cell carcinomas (SCC) and other types of adenocarcinomas. In our study, all patients had abnormal-looking cervixes, but not a high proportion of them had endophytic lesions (17/42, 40.5%), which might be because of a relatively high proportion of early stage diagnoses in our series.

The standard treatment of clinical CCAUC was unclear because of the rarity of this carcinoma. The current treatment strategy mainly refers to the recommendation for SCC. According to the NCCN guidelines, the primary treatment of early stage cervical cancer is either surgery or radical radiotherapy. A retrospective study (15) on early stage cervical cancer showed a poorer 5-year OS in adenocarcinoma than in SCC (*p < 0.05*). An Italian randomized controlled trial (RCT) (16) also showed that adenocarcinoma was an independent poor prognostic factor for OS. The patients with adenocarcinoma who received surgery group had a better PFS and OS than those who received radiotherapy (*p < 0.05*). A large sample analysis of 2,773 early stage adenocarcinomas (17) showed that patients who underwent primary surgery had a better OS (*p < 0.05*). Therefore, adenocarcinoma histology itself was a poor prognostic factor, and primary surgery might be recommended for early stage adenocarcinoma patients. However, the previous studies on early stage CCAUC showed better survivals, which reported that survival rates were between 80% and 91% (4–6). In our study, stage IB to IIA CCAUC patients were performed with radical hysterectomy and showed excellent OS (89.6%). Our data and the previous data all show that CCAUC itself does not appear to exhibit a poor prognosis. We recommend surgery as the primary treatment strategy for the early CCAUC patients. We still need a larger amount of samples and further evaluation about radiation effect in early stage CCAUC.

According to the NCCN guidelines, the patients with IIB stages received primary chemoradiotherapy. Lee DW et al. (18) reported that 192 stage IIB cervical cancer patients were divided into the NACT followed by the surgery group and chemoradiotherapy group, and found that NACT improved the poor pathological prognostic factors, but it did not improve the rate of patient survival compared to the radiotherapy group. Tang J et al. (19) reported on 880 patients with stage IIB-IVA adenocarcinomas and concluded that chemoradiation plus NACT and adjuvant chemotherapy had a lower distant and pelvic failure rate, a better DFS and OS, which might be a very promising treatment protocol for advanced patients. In our study, all eight advanced patients were identified as stage IIB with a poor survival (37.5%), which is consistent with the previous studies of CCAUC (4–6). Among them, four patients (50%) received radiotherapy, three of which (75%) died of the disease, and the other four patients (50%) received NACT followed by surgery, two of which (50%) died of the disease. As our study included only eight stage IIB patients, there were limited evidences for the optimal treatment strategy (NACT followed by surgery or chemoradiotherapy) of patients with CCAUC.

The previous studies (4–6) showed that the 5-year PFS and OS of CCAUC were from 65% to 73% and from 75% to 78%, respectively, and the 5-year PFS and OS of the CCAUC patients with early stage was significantly better than those patients with advanced stage (IIB to IVB). In our study, the 5-year PFS and OS were 68.5% and 77.3%, respectively. The early stage patients (IB to IIA) exhibited a significantly better OS and PFS (*P < 0.05*), which is consistent with the previous finding. Yang L et al. (6) found that advanced tumor stage, large tumor size, and PLN metastasis had a negative effect on PFS and OS of CCAUC patients. Thomas et al. (5) retrospectively analyzed 34 CCAUC patients and revealed that nodal status appears to be a strong predictor of OS and PFS, and they found that positive lymph nodes reduced the PFS and OS. In our study, tumor stage and lymph status significantly effected PFS and OS (p < 0.05). Hanselaar et al. (2) reported that tumor size (>4 cm) was an important negative prognostic factor for CCAUC patients. A retrospective study (20) assessed the 200 early adenocarcinoma (I-IIA) patients who underwent surgery, and found that tumor stage, tumor grade, lymph node status, LVSI, and depth of stromal invasion were significant prognostic variables. In a multivariate analysis, only tumor stage, tumor grade, and nodal status remained significant independent predictors for survival. They showed similar results for survival to the previous studies (21, 22). In our study, the patients with negative PLN had a significantly better 5-year PFS and OS than those with positive PLN (*P < 0.05*). We also analyzed whether those intermediate factors would affect prognosis and found that tumor size (>4 cm), deep stromal invasion, and LVSI did not affect PFS or OS.

Thomas et al. (5) reported that one of eight node-negative CCAUC patients relapsed at 47 months, and they did not recommend the use of RT in node negative patients to improve PFS or OS. Yang L et al. (6) also reported that CCAUC was a radioresistant tumor, and they found that the efficacy of RT or CCRT might be limited for early stage CCAUC patients with risk factors. Our study also revealed that RT or CCRT did not affect PFS or OS in patients with early stage with intermediate factors *(p > 0.05*). At the same time, we revealed that CT did not affect PFS or OS in patients with early stage without risk factors (*p > 0.05*). So we recommend that neither radiotherapy (RT) will be used in early stage patients without high risk factors, nor CT will be used in early stage patients without risk factors.

Some research reported (23–26) that the incidence of ovarian metastasis was 3.7% (range 0% to 12.9%). Ovarian preservation in patients with early stage adenocarcinoma was safe, and had no effect on prognosis. They reminded that large tumor size (>4 cm), deep cervical stromal invasion, lymph node metastasis, corpus uterine invasion, and parametrial invasion were the risk factors of ovarian metastasis. Yang L et al, (6) reported that ovarian metastasis was identified in 1 patient (2.4%) among 41 CCAUC patients who received salping-oophorectomy. Our data showed ovarian metastasis in one patient, which was similar to previous reports. A review (23) including six studies reported that there was no case of ovarian recurrence among more than 100 cases of ovarian preservation in patients with early stage adenocarcinoma, and median follow-up time was 56 months. In our study, we found that one patient with stage IB1 disease who was performed with radical surgery and PLD, without risk factors after surgery, had metastasis of the left ovary at 56 months, and then was performed with surgery and chemotherapy. Now, the patient is still alive with no evidence of recurrence at follow-up time for 80 months. Therefore, the ovarian metastasis rate of early stage CCAUC was not high, but we still recommended prudent ovarian preservation.

Thomas et al. (5) reported that the median time to recurrence was 12 months for CCAUC patients. Reich O et al. (1) reported on 15 CCAUC, 444 SCC, and 59 no-clear cell adenocarcinoma and found that the median time to recurrence was 14 months after the initial therapy, and no significant difference was found among the three groups. Jiang et al. (4) reported that the recurrence time was from 3 months to 31 months. In our study, the median time to recurrence was 19 months (range from 8–56), which is consistent with the previous studies. The long-term follow-up was very important for CCAUC patients.

## Conclusion

In this retrospective study, a series of clinicopathologic factors were analyzed with regard to prognosis. The FIGO stage and pelvic node status were important prognostic factors for both PFS and OS. For treatment modality, we recommended that radical surgery alone will be used in early stage patients without high risk factors, and no further adjuvant RT or CT is required. Ovarian preservation in patients with CCAUC appears to be carefully considered and further explored as the preservation may involve some risk. Although our research was a small-sample retrospective study, we believed that the future application of this information in prospective researches might contribute to improving survival for patients with CCAUC.

## Data Availability Statement

The datasets generated for this study are available on request to the corresponding author.

## Ethics Statement

The studies involving human participants were reviewed and approved by Sun Yat-sen University Cancer Center Research Ethics Committee. Written informed consent for participation was not required for this study in accordance with the national legislation and the institutional requirements.

## Author Contributions

ZL and JLi analyzed the data and wrote the paper. HG, HT, and GL helped in the data collection. JLiu designed the study and supervised the study. All authors contributed to the article and approved the submitted version.

## Conflict of Interest

The authors declare that the research was conducted in the absence of any commercial or financial relationships that could be construed as a potential conflict of interest.
